# Identifying methods to best integrate indigenous knowledge and perspectives within the radiation therapy undergraduate curriculum

**DOI:** 10.1002/jmrs.660

**Published:** 2023-02-13

**Authors:** Thashmira Naidoo, Crispen Chamunyonga, Julie Burbery, Peta Rutledge

**Affiliations:** ^1^ School of Clinical Sciences, Faculty of Health Queensland University of Technology Brisbane Queensland Australia; ^2^ Radiation Oncology Princess Alexandra Hospital Raymond Terrace South Brisbane Queensland Australia; ^3^ Centre for Biomedical Technologies Queensland University of Technology Brisbane Queensland Australia

**Keywords:** Radiation therapy, Indigenous knowledge and perspectives, education, curriculum, cultural safety

## Abstract

The Australian healthcare system continues to work towards close the gap to improve and achieve equality in health and life expectancy for Aboriginal and Torres Strait Islander peoples. When culturally safe practice is forefront, it may be the driving force in improving Indigenous Australian healthcare outcomes. For students and practitioners to be equipped with the industry‐required cultural safety skills, we believe Indigenous Australian knowledge and perspectives must be effectively integrated into undergraduate education. A scoping review of the literature was conducted to identify the most effective teaching and learning methods and assessment tools to best integrate Indigenous knowledge and perspectives into undergraduate radiation therapy curriculum at Queensland University of Technology (QUT). Embase, CINAHL, Scopus and PubMed‐Medline were searched to access peer‐reviewed studies published between 2017 and 2022. A total of 591 articles were identified with 39 full‐text articles meeting the inclusion criteria. Methods of teaching and learning and independent assessment tools that promote cultural capability in undergraduate education were identified. Findings included intensive patient‐specific workshops, which were reported to provide students with an immersive learning experience, better preparing them for clinical placements and future practice. Additionally, other allied healthcare professions have utilised tools such as the Cultural Capability Management Tool (CCMT) and Trans‐Cultural Self‐Efficacy Tool (TSET) to quantitatively assess positive shifts and highlight developmental needs. These findings will inform current educational endeavours to promote cultural safety and the cultural capability continuum in the undergraduate radiation therapy curriculum.

## Graphical abstract

When culturally safe practice is forefront, it may be the driving force in improving Indigenous Australian healthcare outcomes. A scoping review was conducted to identify teaching and learning methods to best integrate Indigenous knowledge and perspectives into undergraduate radiation therapy curriculum. Our findings will inform current educational endeavors, and assist in promoting cultural safety and cultural capability in the undergraduate radiation therapy curriculum.

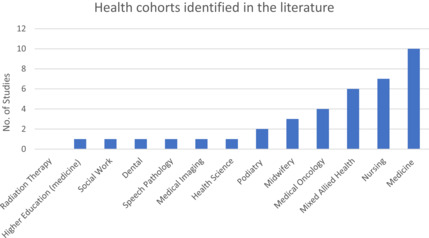

## Introduction

To improve the quality of care for Aboriginal and Torres Strait Islander peoples (hereafter respectfully referred to as Indigenous Australian peoples) and ultimately close the gap, it is imperative to recognise the unique considerations required for this patient demographic.^1^ This requires acknowledging and addressing the determinants of health and their historical causative factors to achieve equitable health care at a practitioner and organisational level.^2^, ^3^ This is highlighted in the Framework for undergraduate health curriculum, and outlined in the Medical Radiation Practice Board of Australia (MRPBA) professional capabilities for medical radiation practitioner registration requirements.^4^, ^5^

Radiation therapists (RTs), alongside radiation oncologists, nurses and other allied health staff, work in a challenging environment to plan and deliver cancer treatments. For RTs to honour the responsibility of perpetuating culturally safe practices and promoting health‐seeking behaviours, foundational education should be well established during undergraduate education.^6^, ^7^, ^8^ Culture should not be considered a disparity in health care; however, it remains to be a primary barrier in preventing Indigenous Australian peoples from seeking and receiving culturally safe care.^9^

Radiation therapy students graduate from university with the requisite knowledge and clinical skills for professional registration; however, the literature suggests a lack of Indigenous health foundational knowledge and subsequent practising confidence among health students and practitioners when required to provide care to Indigenous Australian peoples.^10^, ^11^, ^12^ During a recent review of the undergraduate radiation therapy curriculum at QUT, it was evident that there was a lack of foundational Indigenous health and cultural safety content across the 4‐year degree. Only two of the 16 units in the curriculum addressed Indigenous health or teachings of the cultural continuum, implying better integration of content scaffolded throughout the degree is required. RT students and practitioners should possess an established foundational knowledge of Indigenous Australian history to understand how past and present disparities influence the determinants of health and barriers to accessing culturally safe, responsive health care.^9^, ^13^, ^14^ Therefore, we believe facilitating formal education which promotes cultural capability is key to mitigating the disparities faced and ensuring culturally safe responsive healthcare practices.^8^

Integrating Indigenous Australian knowledge and perspectives into the radiation therapy curriculum is a complex process. Health practitioners need to understand that Indigenous Australian people's health extends well beyond the physical well‐being of the patient. The construct of health is interconnected holistically, socially and spirituality to the whole Indigenous community.^15^ Therefore, the curriculum must be comprehensive and well‐informed to develop students' capabilities for culturally safe practice. Other considerations in the curriculum review process include the MRPBA professional capabilities and accreditation standards for medical radiation practice.^5^, ^15^ Moreover, teaching and learning approaches, as well as assessment methods, must be evidence‐informed and developed to better prepare future practitioners.

The aim of this scoping review was to identify the most effective teaching and learning methods to best integrate Indigenous knowledge and perspectives into tertiary curriculum.

## Methods

A scoping review of the literature was conducted which aimed to identify the most effective teaching and learning approaches and assessment tools for the integration of Indigenous health curriculum. Preferred Reporting Items for Systematic Reviews and Meta‐Analyses (PRISMA) guidelines for scoping reviews were abided by for this review.^16^

### Research question

How can Indigenous knowledge and perspectives be best integrated into the undergraduate radiation therapy curriculum to promote better‐informed practice and cultural continuity?

### Databases and search strategy

Embase, CINAHL, Scopus and PubMed‐MEDLINE were utilised from November 2021–January 2022 to identify studies that reported teaching and learning strategies for Indigenous curriculum in educational and clinical settings. A search strategy was developed with key search terms (Table 1), synonyms and limiters.

**Table 1:** Search terms and synonyms used in the literature search.

Search term	Synonyms
Undergraduate	Higher education OR university OR tertiary
Aboriginal and Torres Strait Islander Peoples	Indigenous Australian Peoples OR Indigenous Australians OR Aboriginal and Torres Strait Islanders OR Aboriginal Australians OR Torres Strait Islanders
Health Education	Education
Cultural capability	Cultural safety OR Cultural continuum OR Cultural competence OR Cultural awareness OR Cultural sensitivity
Integration methods	Delivery mode OR curriculum
Radiation therapy	Undergraduate health OR allied health OR cancer care

### Inclusion and exclusion criteria

The eligibility criteria were determined after defining a PICO question to guide the literature search. Studies were included if they were full text, English language articles that focused on Indigenous Australian people's health knowledge and perspectives, all study types were considered. Studies published from 2017 onwards were sought after to reflect the most current, relevant practices within the cultural continuum – this includes the most correct cultural ‘terminology’ and any advances in practice with Indigenous Australian health. Studies reviewed included undergraduate and qualified practitioner‐based papers.

### Screening

All articles retrieved from the search were exported to Endnote version X9 and assessed for relevance to the scope of the research question. Two authors screened articles for their pertinence to intervention style, learning type or assessment tool – articles that documented knowledge and practising gaps were utilised for the identification of deficits, to help best inform current practice. One author removed duplicates and tabulated summarised findings for analysis. The table was reviewed by a minimum of 2 authors, who also discussed the relevance of the identified studies.

**Figure 1:**
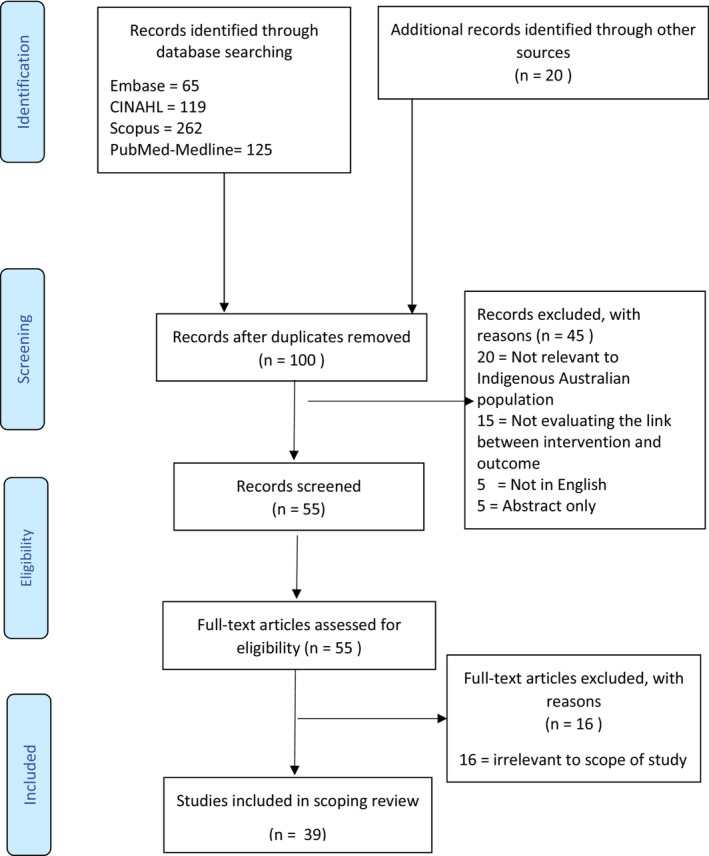
Study flow chart adapted from the Preferred Reporting Items for Systematic Reviews and Meta‐Analyses (PRISMA) diagram.^16^

Our search identified 591 full‐text articles and abstracts, of which 100 were screened after the removal of duplicates. 39 fulfilled the eligibility criteria as seen in the PRISMA diagram (Fig. 1). Studies were assessed for relevance to facilitating Indigenous education in undergraduate cohorts OR outlining literature gaps in this scope of practice.

## Results

### Descriptive analysis

**Figure 2:**
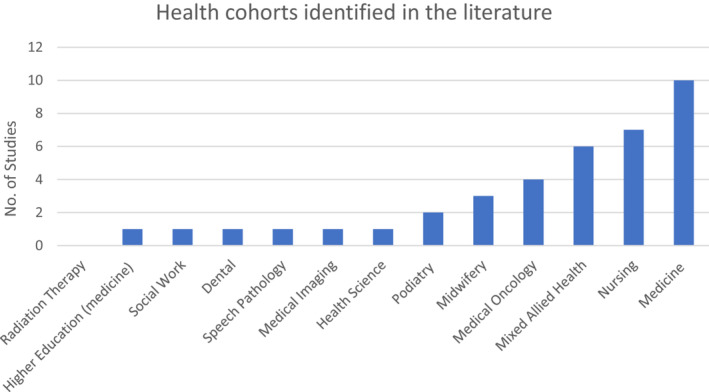
Professional health cohorts investigating Indigenous health and education.

The studies identified highlighted the significant paucity of radiation therapy‐specific literature to facilitating education of Indigenous Australian people's health knowledge and perspectives. As depicted in Figure 2, most studies were from practice‐based medical professions. The cohorts included a mix of undergraduate students and qualified practitioners from the disciples of nursing, midwifery, podiatry, dentistry, medicine and social work.

### Study findings

#### The practice and knowledge deficits

Twenty‐one studies^3^, ^6^, ^9^, ^13^, ^14^, ^15^, ^17^, ^18^, ^19^, ^20^, ^21^, ^22^, ^23^, ^24^, ^25^, ^26^, ^27^, ^28^, ^29^, ^30^, ^31^ were identified that highlighted deficits in level of Indigenous health knowledge which impacted practising standards and confidence in students and qualified practitioners. This was observed and reported through self‐perception confidence tools and workshops, utilised in cohorts of undergraduate and post‐graduate health students and qualified practitioners. Identifying what aspects of practice students and practitioners struggle with was thought to help better consider the most appropriate teaching and learning methods. Table 2 summarises the gaps identified in the literature.

**Table 2:** Practice and knowledge deficits.

Gaps identified	Cohort	Author(s) and year
Indigenous health knowledge deficit	Oncology	Zubrzycki et al., 2017^15^
	Nursing	Power et al., 2021^6^
		Power et al., 2018^17^
	Medicine	Nash et al., 2021^18^
	Midwifery	Thackrah et al., 2020^19^
Unaware of barriers to culturally safe care	Nursing	Withall et al., 2021^20^
Knowledge and cultural safety deficit	Clinical medicine	Ivers et al., 2019^21^
	Podiatry	Gerrad et al., 2021^3^
Cultural un‐awareness	Social work	Fernando et al., 2018^22^
Power differential unawareness	Speech pathology	McDermott et al., 2019^23^
Colonisation knowledge deficit + underconfident practice	Medicine	Yeung et al., 2018^24^
	Health professional educators	Reath et al., 2018^13^
Cultural safety and decolonisation unawareness	Primary health care	Muise, 2019^25^
Inappropriate model of health care	Clinical medicine	Arthur & Rolan, 2019^26^
Unable to link colonisation to determinants of health and health disparities	Clinical medicine	Boffa et al., 2018^27^
		Tujague et al., 2021^14^
Barriers and enablers to accessing culturally safe care	Clinical oncology	Taylor et al., 2021^28^
	Primary health care	Canuto et al., 2018^29^
	Nursing	Li, 2017^9^
Competency vs safety	Clinical medicine	Tujague et al., 2021^14^
	Medical practitioners	Curtis et al., 2019^30^
	Community health	Henderson et al., 2018^31^

The health literature identified an equal number of studies within university cohorts and clinical practice settings. While most studies were from nursing and midwifery, relevant literature was also identified in podiatry, social work, speech pathology and education. Three review articles were also included that commented on cultural competency in the healthcare community.^14^, ^30^, ^31^

#### Delivery of teaching and learning approaches

Investigating modes of curriculum delivery assisted in understanding teaching styles most valued by students. Eighteen studies^2^, ^3^, ^7^, ^11^, ^12^, ^13^, ^20^, ^21^, ^32^, ^33^, ^34^, ^35^, ^36^, ^37^, ^38^, ^39^, ^40^ were identified that directly correlated to a strategy for this outcome (Table 3). Teaching and learning methods were identified both in the context of clinical practice and undergraduate university environments, in equal proportions.

**Table 3:** Teaching and learning methods.

Methods	Observed cohort	Author(s) and year
Intensive workshops	Medicine and nursing	Wilson et al., 2020^13^
	Nursing/midwifery	Withall et al., 2021^20^
	Clinical health practitioners	Durey et al., 2017^32^
Whole school integration – Indigenous Australian curriculum consultant	Nursing and midwifery	Fowler et al., 2021^33^
Progress monitoring	Health students	Mills et al., 2021^7^
Teaching programme	Medicine	Huria et al., 2017^11^
Workshop (physical/online)‐ + Indigenous health unit	Mixed allied health	Mills et al., 2018^2^
		Mills et al., 2022^34^
Transformative learning	Health science	Bullen and Roberts, 2019^12^
	Podiatry	Gerrad et al., 2021^3^
Placement‐oriented	Dental	Mariño et al., 2021^35^
Yarning circles	Medical Imaging	Pilkington et al., 2017^36^
	Midwifery	Fleming et al., 2020^37^
	Clinical services	Ivers et al., 2019^21^
	Clinical oncology	Taylor et al., 2018^38^
	Primary healthcare services	Carlisle et al, 2021^39^
	Survivorship programme	Ristevski et al., 2020^40^
REM framework	Nursing	Power et al., 2018^17^

Abbreviation: REM, Respect, Engagement, Moving Forward.

#### Assessment tools

Three assessment tools, the Cultural Capability Management Tool (CCMT), Trans‐Cultural Self Efficacy Tool (TSET) and a Cultural Competence Clinical Evaluation Tool (CCCET) were identified in the literature (Table 4). These were selected for their relevance to Indigenous Australian content and promotion of the cultural continuum in a controlled, moderated format. These tools offer the opportunity to gauge student cultural capability and awareness levels, monitor progress and serve as quality control for curriculum to inform future development.

**Table 4:** Cultural capability and trans‐cultural self‐efficacy tools.

Assessment Tool	Cohort	Author(s) and year
CCMT	Midwifery	West et al., 2017^8^
	Nursing, midwifery, allied health and biomedical science	West et al., 2018^41^
	Podiatry	West et al., 2021^42^
TSET	Nursing, midwifery, allied health and biomedical science	West et al., 2018^41^
	Nursing	Jeffreys, 2015^43^
CCCET	Nursing	Jeffreys, 2015^43^

Abbreviations: CCMT, Cultural Capability Management Tool; CCCET, Cultural Competence Clinical Evaluation Tool; TSET, Trans‐Cultural Self Efficacy Tool.

## Discussion

The purpose of this scoping review was to identify methods of how to best integrate Indigenous Australian knowledge and perspectives into the undergraduate radiation‐therapy curriculum. The findings were largely collected from Australian university healthcare degrees and clinical settings due to the paucity of radiation therapy‐based studies.

There was significant mention of low levels of Indigenous health knowledge among students and practitioners, along with feeling underconfident when required to provide care to Indigenous Australian peoples, these deficits require further investigation in a subsequent study. Immersive, transformative teaching and learning methods and assessment to specifically aid in increasing student knowledge of Indigenous Australian perspectives and to identify gaps within the undergraduate curriculum, to help fulfil professional registration requirements were identified.

Facilitating cultural education is necessary to ensure culturally capable, responsive healthcare practice.^3^ The identified literature highlights a lack of confidence and application‐based skills when healthcare students and practitioners have to navigate care for Indigenous Australian peoples.^5^, ^11^, ^18^, ^30^, ^32^ Thus, the implementation of Indigenous Australian‐specific intensive workshops and adapted curriculum frameworks have been identified to play a significant role in increasing the knowledge, understanding and practising confidence in undergraduates and practitioners.^11^, ^32^

Learning experiences with Indigenous Australian patient‐specific scenarios would provide students with a safe environment to identify the barriers and enablers to culturally safe care.^13^, ^29^, ^32^ Using patient examples that contrast providing culturally safe, and unsafe care can further consolidate students' understanding and appreciation for identifying these factors.^9^, ^32^, ^33^ Using case studies that address topics such as cancer and the stigma, men's and women's business and patient priorities when regarding family and kin may help develop foundational awareness and considerations.^9^, ^13^, ^28^, ^29^ Durey and colleagues^32^ suggested having a guest Indigenous Australian patient attend a workshop for students to obtain first‐hand experiences of positive and negative patient experiences.^32^ Creating a learning environment where students can say, ‘I don't know what to do’ and equip them with culturally safe, awareness management strategies to enable culturally informed practice is key. Mills and colleagues^7^ highlight the importance of student emotion in transformative learning, and how certain amounts of discomfort contribute to meaningful learning experiences.^7^, ^34^ This is not a ‘one size fits all’ approach by any means. We hope that giving students the opportunities to ask questions, recognise their own level of understanding and problem solve, will consolidate knowledge and encourage culturally safe practice.^18^ The impact of unified culturally safe practice across all health practices can play a significant role in eliminating systemic racism in health systems worldwide.^31^

Fleming and colleagues^37^ reported successful use of yarning circles as professional development for promoting culturally safety.^37^ Yarning or informal chatting with others encourages open, purposeful environments for the exchange of views, which is typically known as a pastime.^18^ Many identified studies^21^, ^36^, ^38^, ^39^ used yarning clinically for Indigenous Australian peoples, reporting an increase in attendance, compliance and removed some ‘shame’ related stigma from diagnosis and side effect associated assumptions.^40^ We believe implementing a yarning workshop for students would increase their knowledge about the practice of yarning and its clinical applications. In creating this space to facilitate open, safe conversations between students and lecturers, we hope to promote cultural awareness from early in the degree.^22^

As awareness is the first step towards becoming culturally safe, we hope this is encouraged with opportunities for reflective practice and challenging discussions regarding power privilege, racism and ongoing disparities.^8^, ^19^, ^22^ Power and colleagues^17^ introduced the REM framework (Respect, Engagement and Moving forward) building on developing awareness so undergraduates can demonstrate cultural respect by graduation.^17^ We hope to adopt this principle of building upon foundational knowledge through course curricula to increase understanding of cultural capability knowledge, consolidated with the aforementioned assessment tools. Lastly, Fowler and colleagues^33^ recommended utilising the services of an Indigenous Australian education consultant to provide course curriculum guidance and quality control. From the identified findings, we believe implementing the workshops, assessment tools and curriculum framework in the undergraduate curriculum would highly benefit student learning and confidence for clinical placements and future practice.^35^

This review identified tools (CCMT, TSET, CCCET) designed for and implemented in podiatry and nursing undergraduates and health practitioners, used to consolidate theoretical knowledge and heighten self‐awareness skills with respect to Indigenous Australian knowledge, cultural safety and clinical practice. These tools are a means of promoting transformative learning aspects, by requiring users to self‐reflect to identify strengths and gaps in Indigenous Australian people's knowledge, therefore increasing self, and cultural awareness.

The CCMT is a cultural capability tool developed based on undergraduate requirements of the National Framework for Aboriginal and Torres Strait Islander Health Curriculum in higher education and assesses the five stipulated domains in achieving cultural capability. These are Respect, Communication, Safety and Quality, Reflection and Advocacy.^8^, ^42^ West and colleagues^8^ demonstrated a positive shift in cultural capability among undergraduate podiatry students when evaluating this tool, indicating use of the CCMT before and after clinical placements is beneficial for the development and awareness of healthcare students with respect to the cultural continuum.^41^, ^42^ While we believe the application of this tool for radiation therapy undergraduates would be highly beneficial, the tool may require adjustment to suit the intended clinical practice scenarios.

The Trans‐Cultural Self‐Efficacy Tool (TSET), which later prompted development of the Cultural Competence Clinical Evaluation Tool (CCCET) are established nursing tools designed and implemented to evaluate undergraduate students' confidence in transcultural nursing skills among diverse patient populations, well suited for the Australian population. The TSET utilises affective (attitudes/values), cognitive (cultural factor knowledge in practice) and practical (confident transcultural communication) subsets to assess self‐efficacy strength and perception and had findings of high content validity and reliability.^41^ The CCCET subsets consist of the provision of cultural‐specific care, cultural assessment and cultural sensitivity to strengthen self‐reflective evaluation and awareness, noted to assist students, teachers and practitioners in recognising their own biases and knowledge deficits.^43^

Findings suggest continuous formative evaluation is required of both students and the curriculum to guide future learning and course development and provide specific improvement of skills to build on the development of professional values as foundational practice to align with registration requirements.^7^, ^42^, ^43^ Implementing such tools during the semester and potentially before and after clinical placements would assist students in performing reflective practice and would help universities track where curriculum gaps occur.^7^, ^41^, ^42^, ^43^ These tools promote self‐reflective practice, essential for this profession and can be used in conjunction with each other and teaching methods to promote cultural capability. These skills help individuals identify, recognise and develop their own biases and assumptions, while identifying gaps in knowledge and set goals for improvement with identified gaps.^12^, ^24^, ^30^, ^35^ Additionally, all tools can serve as a performance indicator or method of quality control for academic staff and universities. This formative assessment would outline baseline levels of knowledge from a student's first year and indicate how that knowledge is built upon through course curriculum and clinical placement.^13^, ^42^ Curriculum must be adapted to continuously challenge students and highlight their privilege, power, biases and beliefs to continue the developmental pathway and formulation of cultural safety to support the self‐determination of Indigenous Australian peoples.^24^, ^25^ Additionally, with the tools assessing specific criteria, this may identify knowledge or content gaps within the curriculum which can be directly addressed.^13^

### Summary and recommendations

Education plays a pivotal role in providing foundational knowledge of Indigenous Australian history in efforts to consolidate culturally capable practice.^10^ This review was part of a comprehensive review of QUTs undergraduate radiation therapy curriculum, to ensure graduates were equipped with the required industry and registration requirements. We identified opportunities for foundational knowledge in fundamental units of study which has been achieved by the inclusion of Indigenous Australian‐specific aetiology, epidemiology and risk factors when learning about different cancers and linking this to the continued health disparities as a consequence of colonisation in treatment‐specific units.^2^ This has now been scaffolded and integrated across all 4 years of the course curriculum. Additionally, we re‐formatted assessment items to include more Indigenous Australian‐specific patient case studies, to increase students' awareness of the barriers and enablers for access to culturally safe care.^13^

We hope to implement intensive workshop sessions that include Indigenous Australian patient case studies, yarning circles and the use of the CCMT, TSET and CCCET for the duration of the course. Future studies may consider developing a radiation therapy‐specific cultural capability assessment tool through further research and adapting the tools as required.

## Conclusion

Closing the gap requires a culturally inclusive undergraduate health curriculum to promote self‐aware, culturally capable graduates and future practitioners.^21^ We identified reputable assessment tools and teaching methods, suitable for integration into the undergraduate radiation therapy curriculum, to promote better‐informed practice and cultural capability. There are practice and knowledge deficits in the literature that necessitate reform of undergraduate curriculum to be more inclusive of Indigenous knowledge and perspectives, not only for radiation therapy but also all allied health degrees. Future research should focus on designing degree‐specific cultural capability tools, and further investigate the cultural continuum specific to radiation therapy.

## Conflicts of Interest

The authors declare no conflict of interest.
